# Digestibility of Natural and Recombinant Allergenic Peanut Proteins in Artificial Gastrointestinal Fluids

**DOI:** 10.14252/foodsafetyfscj.D-25-00016

**Published:** 2025-12-19

**Authors:** Mizuho Terashima, Rina Matsuoka, Takumi Nishiuchi, Hiroaki Kodama, Taira Miyahara

**Affiliations:** 1 Graduate School of Horticulture, Chiba University, 1-33 Yayoi-cho, Inage-ku, Chiba 263-8522, Japan; 2 Division of Biological Science and Technology, Graduate School of Natural Science and Technology, Kanazawa University, Kanazawa 920-1192, Japan; 3 Division of Integrated Omics research, Bioscience Core Facility, Research Center for Experimental Modeling of Human Disease, Kanazawa University, Kanazawa 920-8640, Japan; 4 Sapiens Life Sciences, Evolution and Medicine Research Center, Kanazawa University, 13-1 Takaramachi, Kanazawa, Ishikawa, 920-8640, Japan

**Keywords:** allergenic protein, Ara h 1, Ara h 2, digestibility test, newly expressed proteins, peanut

## Abstract

Safety assessments are necessary for genetically modified foods in many countries, including Japan. Stabilities during pepsin, trypsin, or pancreatin digestion are a key criterion for assessing the allergenic potential of newly expressed proteins (NEPs). In digestibility tests, NEPs produced by heterologous expression systems were frequently used. Polyhistidine tags (His-tags) are primarily/often used to purify recombinant proteins. Studies of His-tags’ influences remain limited on the susceptibility of a protein to pepsin/trypsin digestion, although His-tags can affect protein folding and stability. In this study, we compared the digestibility of the natural peanut allergenic proteins Ara h 1 and Ara h 2 to the recombinant Ara h 1 protein with N-terminal His-tag and recombinant Ara h 2 protein with C-terminal His-tag, respectively. Peptides after the proteolysis were then analyzed using liquid chromatography–tandem mass spectrometry to determine the proteolytic cleavage sites. Differences were detected in the C-terminal region after pepsin cleavage of the His-tag extension of Ara h 1 and Ara h 2 proteins. No differences were observed in other cleavage sites between the natural and recombinant Ara h 1 and Ara h 2 proteins. The N-terminal region of Ara h 1 and Ara h 2, at which the epitopes recognized by most patients allergic to peanut were located, was equally resistant to pepsin digestion regardless of the natural or recombinant forms. In this study, an unintended short protein isoform was detected in the recombinant Ara h 2 samples. This short recombinant isoform may be misfolded, and it showed reduced susceptibility to pepsin digestion relative to natural full-length Ara h 2. In this short Ara h 2 isoform, newly paired disulfide bonds may make it more rigid. Recombinant proteins with His-tags can provide nearly comparable results to the corresponding natural proteins in protease digestions and thus offer information useful for safety assessment.

## 1. Introduction

The commercialization of genetically modified (GM) crops began in the 1990s. At present, the approval of commercial GM crops remains subject to stringent regulatory frameworks. The Codex Alimentarius guidelines established fundamental regulatory frameworks for risk assessment of GM foods^1^^)^, and assessments of the allergenic potential of newly expressed proteins (NEPs) are of great importance. These assessments generally comprise a history of the safe use of NEPs, bioinformatic analyses designed to identify potential hazards by determining the similarity to known allergens, protein stability assessments following pepsin digestion in simulated gastric fluid (SGF) and pancreatin/trypsin digestion in simulated intestinal fluid (SIF), and protein stability during the processing of grains into food and feed products. This processing includes cooking phases; thus, heat stability is included as an additional assessment criterion^2^^,^^3^^)^. Moreover, when the safe use of NEPs cannot be substantiated on the basis of the abovementioned items, performing serological analysis, such as human serum IgE binding to NEPs, is necessary.

SGF/SIF digestibility tests should be conducted using NEPs prepared from GM crops. However, NEPs within crops are usually of low concentrations, and their purification from grains is laborious. Therefore, NEPs heterologously produced by *Escherichia coli* and yeast expression systems are commonly used during SGF/SIF digestibility testing^4^^)^. Protein folding markedly affects protein digestibility, particularly during SIF digestibility testing^5^^)^. For example, tightly and properly folded proteins exhibit resistance to SGF/SIF digestion, whereas those undergoing denaturation protocols such as heat treatment usually exhibit enhanced digestibility. Consequently, the use of incorrectly folded proteins during SGF/SIF digestibility testing can influence the assessment of allergenic potential. Moreover, in heterologous expression systems, overexpressed heterologous proteins can be misfolded and, therefore, can be subjected to proteolytic degradation^6^^)^. The tag sequences are frequently used to facilitate the purification of heterologously expressed NEPs. In general, polyhistidine tags (His-tags) are used for the purification of recombinant proteins. Despite the small size (approximately 2.5 kDa) and limited effect of His-tags on the structure of recombinant proteins^7^^)^, several studies have shown the distinctive effects of His-tags on protein function and stability. For example, reduced enzymatic activities caused by the addition of His-tags were observed in the recombinant trehalose synthase^8^^)^ and chlorocatechol 1,2-dioxygenase^9^^)^. Of the 10 different proteins studied, the thermal stability of six proteins showed a change in stability, which was related to the presence or absence of the His-tags^10^^)^. Therefore, conformational differences caused by the addition of His-tags to heterologously expressed proteins may result in variations in the access of the digestion enzymes to potential cleavage sites during SGF/SIF digestibility testing.

Continuous monitoring of allergy symptoms following the consumption of peanuts (*Arachis hypogaea*) is essential because of the persistent nature of peanut allergy into adulthood^11^^)^. Peanuts contain 18 allergenic proteins (http://www.allergen.org/), with Ara h 1 being the most abundant^12^^)^. Ara h 1 is a homotrimer of a 63 kDa protein containing a cupin motif^13^^,^^14^^,^^15^^)^. Ara h 2 is the most potent allergen, and it strongly triggers mast cell activation^16^^,^^17^^)^. The Ara h 2 protein is a 2S albumin with a molecular mass of 17 kDa^18^^,^^19^^,^^20^^)^. Ara h 1 and Ara h 2 exhibit resistance to degradation by digestive enzymes^21^^)^. In this study, these two proteins were used as a model for studying the differences in the digestibility of recombinant proteins with His-tags and natural proteins. Specific peptides that were generated following pepsin or trypsin digestion of natural and recombinant Ara h 1 and Ara h 2 proteins were characterized using liquid chromatography–tandem mass spectrometry (LC–MS/MS), thereby allowing us to compare the digestibility of natural and recombinant proteins with His-tags. The results indicate that the use of recombinant proteins with His-tags can provide nearly comparable results to the corresponding natural proteins for safety assessment, although His-tag extension partially affected the protease digestibility.

## 2. Materials and Methods

### 2.1 Allergenic Peanut Proteins

Allergenic peanut proteins were obtained as follows: natural Ara h1 protein (nAra h 1, code no. LTN-AH1-1, InBio, VA, USA) and natural Ara h 2 protein (nAra h 2, code no. NA-AH2-1, InBio) were prepared from peanut seeds. Recombinant Ara h1 protein with N-terminal His-tag was produced using recombinant *Escherichia coli* (rAra h 1, code no. CSB-EP331779ANE(A4), Cusabio, TX, USA), whereas recombinant Ara h 2 protein with C-terminal His-tag was produced from *Pichia pastoris* expressing Ara h 2.0201 cDNA (rAra h 2, code no. RP-AH2-1, InBio).

### 2.2 SGF Digestibility Test

Two types of porcine pepsin were purchased from Sigma-Aldrich (S-pepsin, code no. P7000, MO, USA) and Promega (P-pepsin, code no. V1959, WI, USA). Prior to testing, each type of pepsin was dissolved to a final concentration of 2.88 µg/µL in SGF buffer (0.07 M HCl, 0.03 M NaCl, pH 2.0). The digestibility test reaction mixtures consisted of SGF buffer, pepsin, and Ara h 1 or Ara h 2 in a total volume of 20 µL. The proportion of pepsin to protein was as indicated in the respective figure legends. For each assay, the mixture pH was adjusted to 1.7–2.0. During testing, the reaction mixtures were incubated for 5 or 60 min (depending on the experiment) at 37°C, after which the reaction was stopped by adding 10 µL of 0.1 M Na_2_CO_3_. Otherwise, no reducing agents or protein denaturants were used during digestion.

### 2.3 Successive Digestion Using Pepsin and Pancreatin

A successive digestion was tested as follows: 15 μg of nAra h 1 was digested with S-pepsin (Sigma-Aldrich) in a 20-μL reaction mixture for 5 min. The pepsin digestion reaction was stopped by adding 5 μL of 0.2 M Na_2_CO_3_ and 5 μL of distilled water. Next, porcine pancreatin (code no. P7545, Sigma-Aldrich) was dissolved to a final concentration of 90 µg/µL in SIF buffer (0.05 M KH_2_PO_4_; 0.02 M NaOH, adjusted to pH 7.0). For subsequent digestion with pancreatin, 5 μL of the pepsin-digested solution was mixed with 22 μL of SIF buffer and 3 μL of pancreatin. Then, the resulting mixture was incubated at 37°C for 120 min, after which the reaction was stopped by cooling on ice. Finally, 17.5 μL of the successively digested solution was subjected to LC–MS/MS.

### 2.4 SIF Digestibility Test Using Trypsin

Porcine trypsin (code no. 90057, Thermo Fisher Scientific, MA, USA) was dissolved to a final concentration of 0.03 µg/µL in SIF buffer. Then, 20 µL of reaction mixtures, containing 3.0 µg of peanut proteins, 2 µL of trypsin, and 16 µL of SIF buffer, were created. These mixtures were incubated at 37°C for either 60 or 120 min, after which the reactions were stopped by adding 10 µL of deionized water and the tubes were immediately cooled on ice.

### 2.5 Reduction of Disulfide Bonds in Ara H 2 protein with Subsequent Pepsin Digestion

In this study, dithiothreitol (DTT) was used to reduce the disulfide bonds in the Ara h 2 proteins. The reduction protocol was as follows: 1.5 µg of rAra h 2 was initially dissolved in 10 mM DTT before being incubated at 50°C for 5 min. Then, the Ara h 2 mixture was mixed by tapping the tubes, which were further incubated for another 5 min at 50°C. Subsequently, the rAra h 2 solutions were used for digestion with P-pepsin. Considering that DTT can slightly inhibit pepsin activity, the concentration of DTT in the pepsin digestion mixture was diluted to 1.2 mM. Overall, the pepsin digestion mixture consisted of 1.5 µg of DTT-treated rAra h 2 and 1 µg of P-pepsin in a 10-µL SGF solution (pH 1.5) reaction mixture, which were incubated at 37°C for 60 min.

### 2.6 Sodium Dodecyl Sulfate-polyacrylamide Gel Electrophoresis (SDS-PAGE)

Samples for SDS-PAGE were prepared as follows: 2.5 µL of 6× sample buffer (code no. 09499-14, Nacalai Tesque, Kyoto, Japan) was added to 12.5 µL of the digested protein solution. Then, the resulting mixture was heated at 95°C for 5 min before being cooled to ambient temperature. Then, the solutions were loaded onto a 5%–20% Bullet PAGE Plus Precast Gel (code no. 21797-24, Nacalai Tesque), after which the proteins were separated in running buffer (code no. 30329-61, Nacalai Tesque) at 10 mA for 15 min and then 30 mA for 20 min. The Precision Plus Protein Dual Xtra Prestained Protein Standards (code no. 1610377, Bio-Rad Co., CA, USA) were used as molecular weight markers. After separation, the gels were washed three times with deionized water before being stained with a Coomassie brilliant blue staining solution (code no. SP-4010, Apro Science Co., Tokushima, Japan). All procedures were performed in accordance with the manufacturer’s protocol. Finally, the gels were imaged using a Gel Doc EZ Imager (Bio-Rad).

### 2.7 Peptide Sequencing by Edman Degradation

For peptide sequencing, the rAra h 2 protein and its short isoform were initially separated by SDS-PAGE before being transferred onto a PVDF membrane. Then, the proteins on the membrane were visualized by staining with amidoblack, after which a portion of the membrane containing the rAra h 2 short isoform was excised. Subsequently, the peptide sequences were determined by the facilities at the Division of Instrumental Analysis, Okayama University (Okayama, Japan).

### 2.8 LC–MS/MS Analysis

For LC–MS/MS, 17.5 µL of digestion samples was initially transferred to an ultrafiltration tube (Amicon Ultra Centrifugal Filter, 3 kDa MWCO, MERCK, Darmstadt, Germany) before being centrifuged at 500 *g* for 30 min. Then, the resulting filtrates were used for LC–MS/MS analysis. Subsequently, the digested peptides were purified using a strong cation exchange capillary column (GL Sciences Inc., Tokyo, Japan), with all procedures following the manufacturer’s protocol. Next, the peptides were desalted using a stage tip #84850 (Thermo Pierce, Tokyo, Japan) before being eluted with 70% acetonitrile (ACN). Subsequently, the eluted peptides were subjected to vacuum centrifugation to remove the residual solvent and then solubilized in 5% ACN containing 0.1% trifluoroacetic acid. Next, the purified peptides were added to an Aurora column (25 cm × 75 μm ID, 1.6 mm C18; IonOptics, Fitzroy, Austria) and separated using a linear ACN gradient (0–40%) in 0.1% formic acid at a flow rate of 300 nL min^−1^. Then, the peptide ions were identified by using an Orbitrap QE plus MS (Thermo Fisher Scientific) in a data-dependent acquisition mode as implemented in Xcalibur version 4.4 (Thermo Fisher Scientific, San Jose, CA, USA). Full-scan mass spectra were acquired via MS over the range of 375 to 1,500 m/z with a resolution of 70,000 m/z. All raw data files were processed using PEAKS studio 10 (Bioinformatics Solutions Inc., Waterloo, Canada). Next, *de novo *peptides were identified using a precursor mass tolerance of 10 ppm, a fragment ion mass tolerance of 0.02 Da, and strict trypsin specificity, which allowed up to two missed cleavages. Finally, the PTM mode was used to map the peptide sequences to the protein sequences of Ara h 1 (Uniprot accession: P43237) and Ara h 2 (Uniprot accession: Q6PSU2), respectively, including approximately 200 posttranslational modifications.

## 3. Results

### 3.1 Partial Pepsin Digestion of Ara H 1

**Fig. 1.**
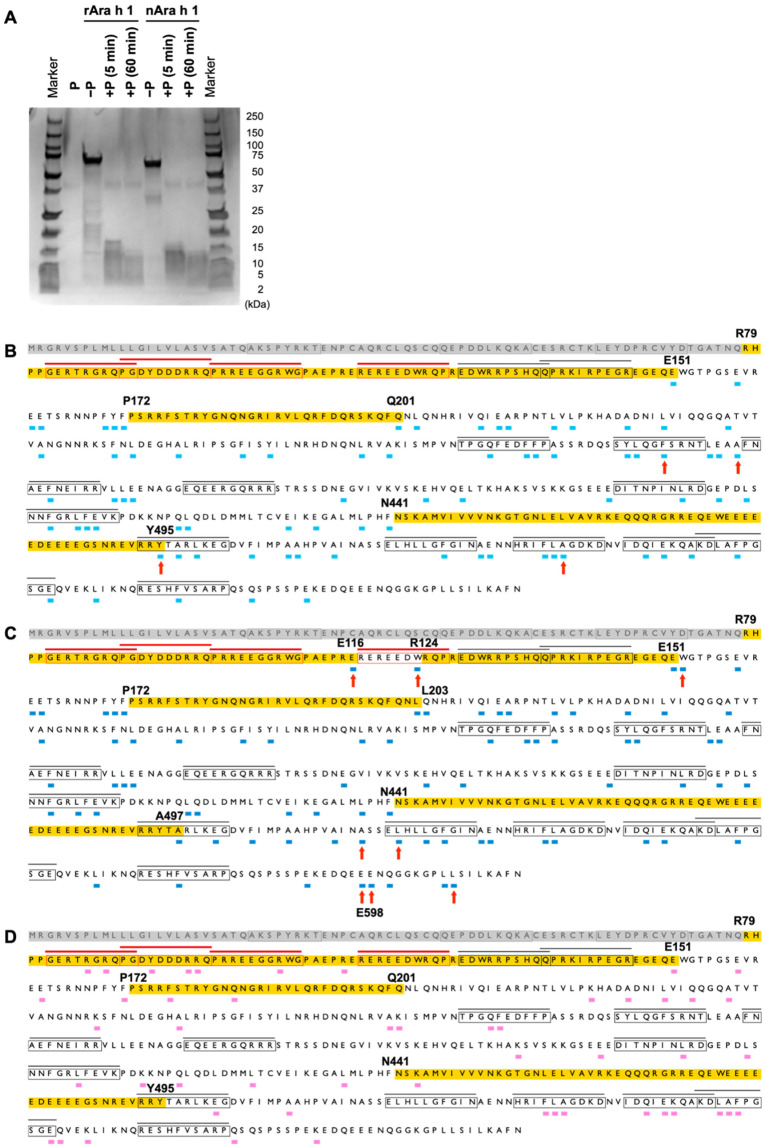
Profiles of the Ara h 1 protein fragments generated from S-pepsin digestion and successive digestion with S-pepsin and pancreatin. The amino acid sequences shown in the red and black boxes indicate epitopes. In providing a comprehensive understanding of the epitope region, the epitopes are shown in red and black lines on the corresponding amino acid sequences. The peptides shown in red boxes and red lines represent public epitopes. The deduced cleavage sites were mapped, and they are shown in short lines under the amino acid sequence of the Ara h 1 protein. (A) SDS-PAGE profile of Ara h 1 proteins digested with S-pepsin. P indicates S-pepsin. (B) Cleavage sites generated by nAra h 1 following 60-min digestion with S-pepsin. (C) Cleavage sites generated by rAra h 1 following 60-min digestion with S-pepsin. The amino acid sequences highlighted in yellow indicate the regions in which no cleavage sites were detected and have >28 amino acids. The arrows indicate the cleavage sites exclusively detected in nAra h 1 or rAra h 1 proteins. (D) Cleavage sites generated by nAra h 1 following 5-min digestion with S-pepsin and 120-min digestion with pancreatin. The ratio of Ara h 1 to S-pepsin was 0.52:1 (A–D), and that of Ara h 1 to pancreatin was 0.0093:1 (D). In Fig. 1D, yellow lines highlight the same region as those in Fig. 1B.

First, the pepsin digestion of two differently prepared Ara h 1 proteins was analyzed. One was a natural form of Ara h 1 (nAra h 1, InBio) prepared from peanut extract, whereas the other was a recombinant form of Ara h 1 (rAra h 1, CUSABIO) produced heterologously by *E. coli* cells. The molecular mass of nAra h1 is 64.5 kDa^22^^)^, whereas that of rAra h 1 with a His-tag is 71.7 kDa; these values were provided by their respective manufacturers. The corresponding molecular masses of nAra h 1 and rAra h 1 were confirmed by SDS-PAGE (**Fig. 1A**). Under nondenaturing conditions, rAra h 1 exists as a trimer^23^^)^, whereas nAra h 1 can be purified as an oligomer^24^^)^. The oligomeric structure of Ara h 1 was revealed by native-PAGE, which revealed the estimated molecular masses of nAra h 1 and rAra h 1, indicating that both allergens were present as trimers (**Fig. S1**).

The nAra h 1 and rAra h 1 proteins were digested with S-pepsin (Sigma-Aldrich Co.) for 5 and 60 min, respectively, resulting in similar digested fragments from both proteins. After a 60-min digestion, nAra h 1 and rAra h 1 were degraded into fragments smaller than 15 kDa (Fig. 1A). Then, these digested products were subjected to LC–MS/MS analysis, revealing peptides containing 7–25 amino acids. Overall, approximately 50–70 unique peptides were identified by LC–MS/MS from samples digested for 5 or 60 min with S-pepsin (**Table S1**). The C-terminal amino acid residues of the digested peptides were identified as the cleavage sites (**Fig. S2**). The cleavage sites generated by the 5-min (**Fig. S3**) and 60-min (Fig. 1B, C) digestion were mapped onto the full Ara h 1 amino acid sequence. Ara h 1 is translated as a pre-pro-protein, and two-step cleavage is necessary to generate a mature Ara h 1^25^^)^. Since the 79-amino-acid peptide present at the N-terminal was removed during the maturation of Ara h 1, this N-terminal region was excluded from the mapping of cleavage sites. Based on the results obtained by S-pepsin digestion analysis, four cleavage sites were detected exclusively in nAra h 1, whereas eight were observed only in rAra h 1 (Figs. 1 and **S3**). The remaining pepsin cleavage sites were observed in both nAra h 1 and rAra h 1.

Next, continuous amino acid stretches longer than 28 amino acids were identified in which no cleavage sites were observed. Three of these chains, namely, R79 to E151, P172 to Q201/L203, and N441 to Y495/A497, were identified as potential digestion-resistant regions. LC–MS/MS analysis cannot detect peptides shorter than four amino acids in length. Thus, if a pepsin-sensitive region undergoes digestion into fragments smaller than four amino acids, the cleavage sites responsible cannot be mapped. To clarify whether these three regions were resistant or sensitive to pepsin digestion, the nAra h 1 protein was initially digested with S-pepsin for 5 min and then subjected to a successive digestion with pancreatin for another 120 min. Consequently, multiple peptides were generated from the regions of R79 to E151 and P172 to Q201/L203. However, only one peptide was generated from the N-terminal region of N441 to Y495/A497 (Fig. 1D). Therefore, at least two regions, from R79 to E151 and from P172 to Q201/L203, are resistant to pepsin digestion.

### 3.2 Complete Pepsin Digestion of Ara H 1

**Fig. 2.**
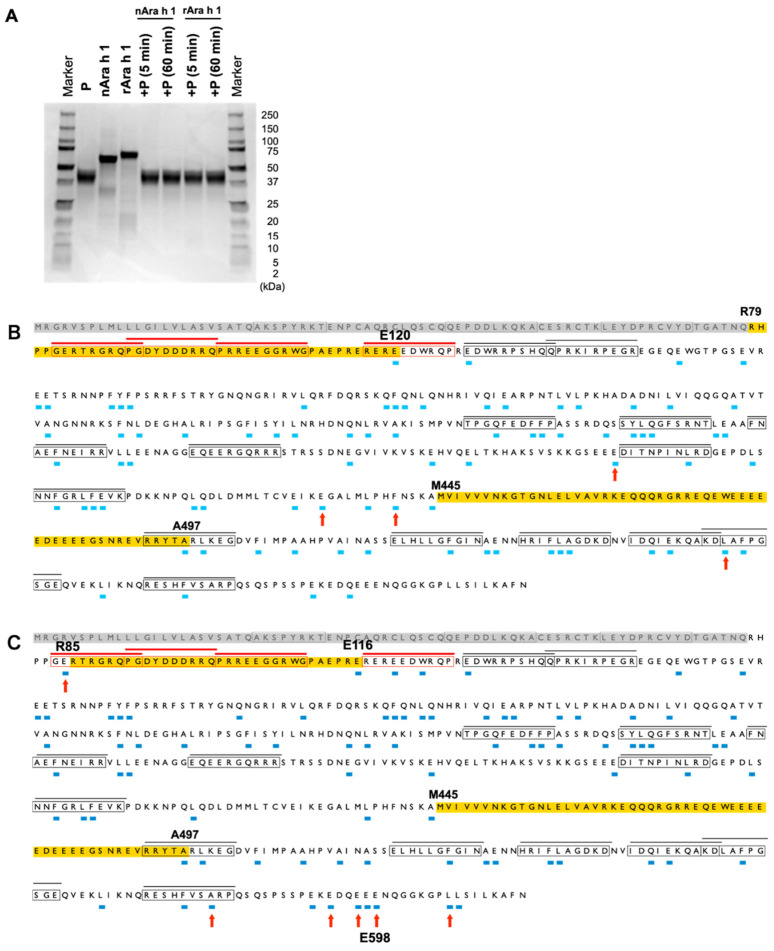
Profiles of the Ara h 1 protein fragments generated from P-pepsin digestion. (A) SDS-PAGE profile of the P-pepsin-digested Ara h 1 proteins. P indicates P-pepsin. (B) Cleavage sites generated by nAra h 1 following 5-min digestion. (C) Cleavage sites generated by rAra h 1 following 5-min digestion. The ratio of Ara h 1 to P-pepsin was 0.52:1 (A–C). The amino acid sequences highlighted in yellow indicate the regions in which no cleavage sites were detected and have >28 amino acids. Arrows indicate the cleavage sites that were exclusively detected in the nAra h 1 or rAra h 1 proteins. The epitopes and cleavage sites are shown as described in the legend of Fig. 1.

Next, the peptides generated by complete pepsin digestion of the Ara h 1 proteins were characterized by using pepsin from Promega (P-pepsin), which has an approximately twofold higher specific activity relative to S-pepsin (**Fig. S4**). Moreover, high concentrations of P-pepsin were used to achieve complete digestion. After 5-min digestion with P-pepsin, nAra h 1 and rAra h 1 were completely digested (**Fig. 2A**). Next, the peptides generated by the 5-min (**Fig. 2**) and 60-min (**Fig. S5**) digestion were identified, and their respective cleavage sites were mapped. As shown in **Fig. 1**, the susceptibility of a region from N441 to Y495/A497 to S-pepsin digestion remains to be elucidated. After a 5-min digestion with P-pepsin, no peptides were detected from this region in either nAra h 1 or rAra h 1 (**Fig. 2**). However, when the nAra h 1 protein was digested with P-pepsin for 60 min, no cleavage sites were identified in the region from N441 to A497, although one peptide was generated from the corresponding region of the rAra h 1 protein following 60-min digestion (**Fig. S5**). If the fragment harboring the region from N441 to A497 is resistant to pepsin digestion, then an approximately 6-kDa fragment will be generated. However, considering that SDS-PAGE showed the complete digestion of Ara h 1 proteins (**Fig. 2A**), the region from N441 to A497 may be susceptible to pepsin digestion. Another notable observation from the S-pepsin and P-pepsin treatments was that the cleavage sites exclusively identified in the rAra h 1 protein were preferentially located at the C-terminal region (Figs. 1C and2C). For example, S-pepsin and P-pepsin cleaved at E598 of rAra h 1 but not in nAra h 1. Therefore, the folding of the C-terminal region of rAra h 1 may differ from that of nAra h 1.

### 3.3 Trypsin Digestion of Ara H 1 proteins

**Fig. 3.**
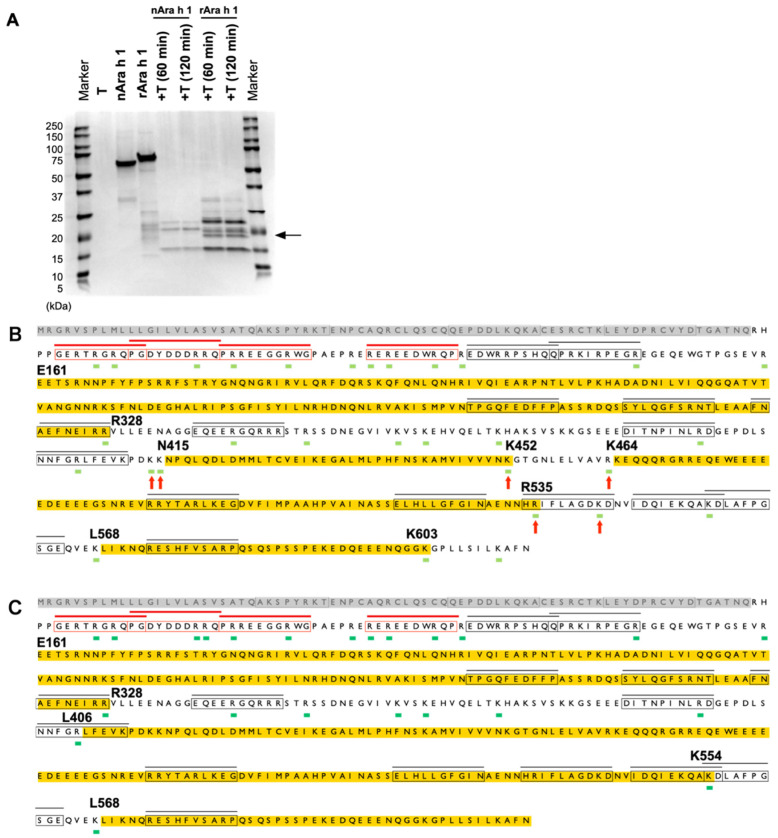
Profiles of the Ara h 1 protein fragments generated from trypsin digestion. (A) SDS-PAGE profile of trypsin-digested Ara h 1 proteins. T indicates trypsin. (B) Cleavage sites generated by nAra h 1 following 120-min digestion. (C) Cleavage sites generated by rAra h 1 following 120-min digestion. The ratio of Ara h 1 to trypsin was 50:1 (A–C). The amino acid sequences highlighted in yellow indicate the regions in which no cleavage sites were detected and have >28 amino acids. The arrows indicate the cleavage sites that were exclusively detected in the nAra h 1 protein. The epitopes and cleavage sites are shown as described in the legend of Fig. 1.

Pancreatin comprises several enzymes, including amylase, lipase, and protease. Therefore, the SDS-PAGE profiles of the pancreatin-digested proteins showed complex patterns. In identifying the degraded polypeptides, trypsin, which is a prominent digestive enzyme present in pancreatin, was used in all subsequent experiments. The trypsin digestion of Ara h 1 was conducted independently in duplicate (Figs. 3 and** S6**). SDS-PAGE analysis revealed that the digestion of the nAra h 1 and rAra h 1 proteins generated three and four major fragments, respectively (**Fig. 3A**). For example, an 18-kDa fragment was generated preferentially by trypsin-digested rAra h 1. Then, the cleavage sites generated during the 120-min trypsin digestion were mapped. Compared with pepsin digestion, the N-terminal pepsin-resistant region (i.e., R79 to E151; **Fig. 1**) was readily digested by trypsin (**Fig. 3B, C**). Based on the two datasets obtained from two independent, duplicate experiments (Figs. 3 and **S6**), six cleavage sites that were exclusive to nAra h 1 were identified, and the generation of two shorter nAra h 1 fragments was deduced (i.e., N415 to K452 and K464 to R535). By contrast, long trypsin-resistant rAra h 1 fragments (i.e., L406 to K554, **Fig. 3**; H365 to K554, **Fig. S6**) were deduced from trypsin-digested rAra h 1. Overall, the differences in cleavage sites between nAra h 1 and rAra h 1 may account for the generation of the 18-kDa rAra h 1 fragment observed by SDS-PAGE.

### 3.4 Pepsin Digestion of Ara H 2

**Fig. 4.**
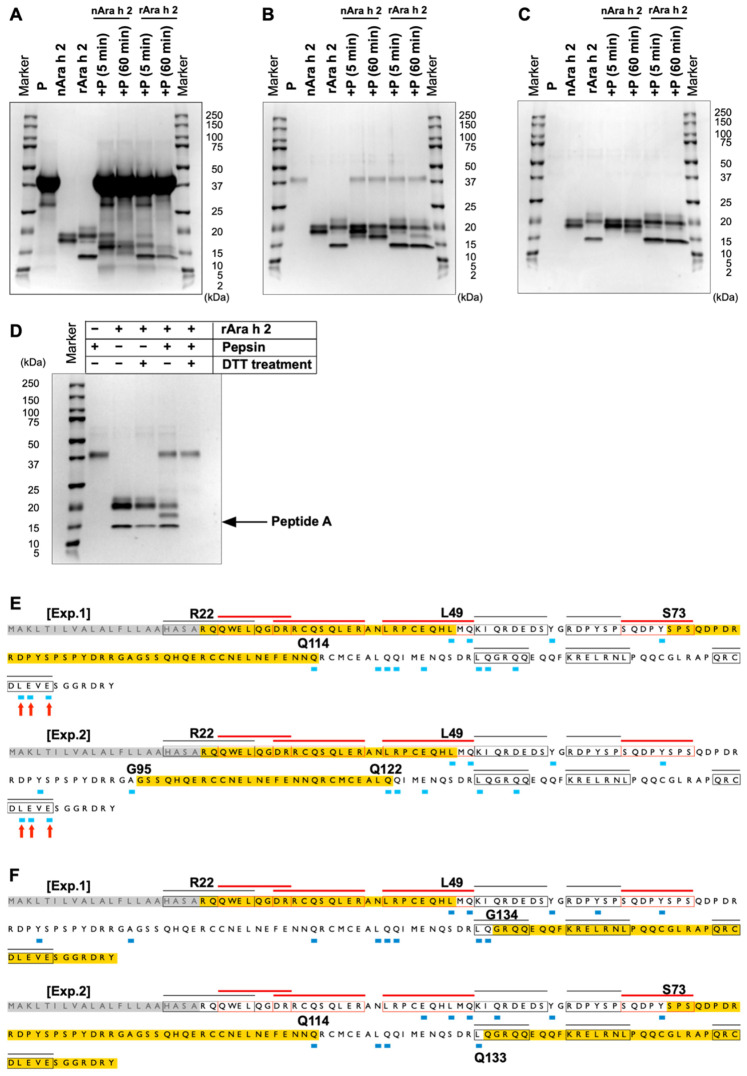
Profiles of the Ara h 2 protein and DTT-treated rAra h 2 protein fragments generated from P-pepsin digestion. (A, B, C) SDS-PAGE profiles of Ara h 2 proteins digested with P-pepsin. The ratios of Ara h 2 to P-pepsin were 0.052:1 (A), 5.2:1 (B), and 52:1 (C). P indicates P-pepsin. (D) SDS-PAGE profile of the digested products of DTT-treated rAra h 2 proteins. Aliquots of rAra h 2 were initially treated with DTT and then digested with P-pepsin. The ratio of rAra h 2 to P-pepsin was 1.5:1. (E) Cleavage sites generated from nAra h 2 following 5-min digestion with P-pepsin. (F) Cleavage sites generated from rAra h 2 following 5-min digestion with P-pepsin. The results of two independent experiments are also shown. The ratio of Ara h 2 to P-pepsin was 0.052:1. The amino acid sequences highlighted in yellow indicate the regions in which no cleavage sites were detected and have >28 amino acids. The arrows indicate the cleavage sites that were exclusively detected in the nAra h 2 protein. The epitopes and cleavage sites are shown as described in the legend of Fig. 1.

The natural Ara h 2 protein (nAra h 2, Sigma-Aldrich) comprises Ara h 2.01 and Ara h 2.02, with molecular masses of 16.3 and 18 kDa, respectively^20^^,^^26^^)^. Ara h 2.01 lacks a region from Q76 to S87 (UniProt Q6PSU2 Sequence & Isoforms), which generates two distinct bands that were visible by SDS-PAGE analysis of nAra h 2. By contrast, recombinant Ara h 2 (rAra h 2, Sigma-Aldrich) has a His-tag at its C-terminus. Overall, full-length rAra h 2 was detected at a slightly higher migration position than nAra h 2 (**Fig. 4A**). In addition, a short rAra h 2 isoform with a molecular mass of 13.5 kDa was observed. The N-terminal region of this isoform was identified through Edman degradation, which corresponded to the fragment from G93 to the C-terminal amino acid, including all His-tag residues. This fragment is hereafter referred to as peptide A.

Considering that Ara h 2 is resistant to pepsin digestion^27^^)^, P-pepsin was used, which has a relatively high specific activity (**Fig. S4**). Accordingly, nAra h 2 and rAra h 2 were digested with three concentrations of P-pepsin. The Ara h 2:P-pepsin ratios were 0.052:1 (Fig. 4A), 5.2:1 (Fig. 4B), and 52:1 (Fig. 4C). When digestion was conducted for 60 min under a high concentration of pepsin (Fig. 4A), full-length nAra h 2 and rAra h 2 disappeared, and small fragments with a molecular weight <10 kDa were generated. Moreover, partially degraded fragments were produced in a similar manner by the nAra h 2 and rAra h 2 samples.

Then, the peptides generated by digestion with high concentrations of pepsin (for 5 and 60 min) were identified by LC–MS/MS (**Table S2**). Next, their cleavage sites were mapped onto the Ara h 2 amino acid sequence. The cleavage sites generated by the 5-min digestion are shown in Fig. 4, and those generated by the 60-min digestion are shown in **Fig. S7**. Considering that the 21-residue N-terminal sequence is a signal sequence^28^^)^, this region was excluded from peptide mapping. Notably, three cleavage sites were exclusively observed in the C-terminal region following the 5- and 60-min digestion of nAra h 2 (Figs. 4E and **S7**). Therefore, the His-tag sequence present at the rAra h 2 C-terminus may influence pepsin digestibility. Peptide A (i.e., the sequence from G93 to the C-terminal amino acid) exhibited resistance to pepsin digestion and persisted even after 60 min of pepsin digestion (Fig. 4A). However, many peptides were generated from a region spanning Q114 to the C-terminus following pepsin digestion. This result indicates that in the full-length Ara h 2 protein, the region corresponding to peptide A exhibits greater susceptibility to pepsin digestion than peptide A. Therefore, the conformation of peptide A may be distinct from that of the corresponding region of full-length Ara h 2.

In addition, Ara h 2 possesses several disulfide bonds that are crucial for folding and stability^29^^)^. These disulfide bonds render the polypeptides inaccessible to proteolysis^15^^,^^30^^)^. In this study, the disulfide bonds of rAra h 2 were reduced by DTT treatment, and the resultant rAra h 2 was digested with pepsin. Notably, DTT-treated rAra h 2 and peptide A were completely digested (Fig. 4D**)**. Therefore, the stability against pepsin digestion was dependent on the disulfide bonds present in the Ara h 2 protein.

### 3.5 Trypsin Digestion of Ara H 2 proteins

**Fig. 5.**
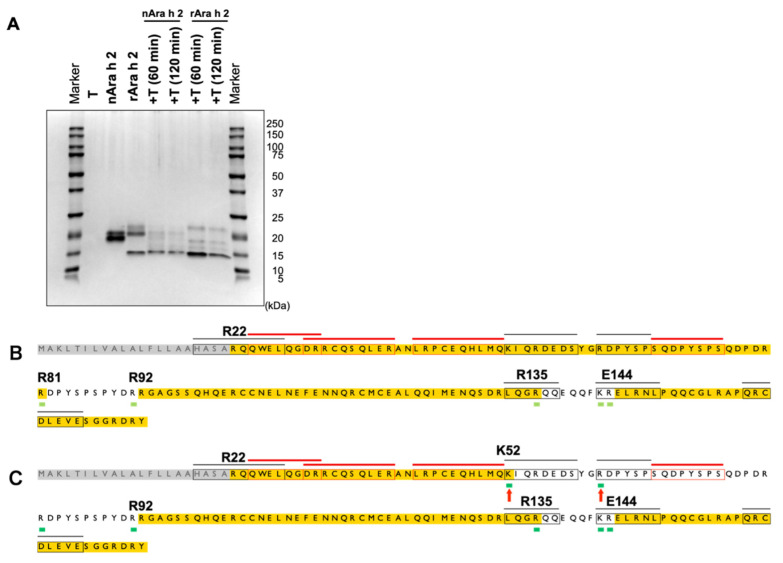
Profiles of the Ara h 2 proteins generated from trypsin digestion. (A) SDS-PAGE profile of trypsin-digested Ara h 2 proteins. T indicates trypsin. (B) Cleavage sites generated by nAra h 2 following 120-min digestion. (C) Cleavage sites generated by rAra h 2 following 120-min digestion. The ratio of Ara h 2 to trypsin was 50:1 (A–C). The amino acid sequences highlighted in yellow indicate the regions in which no cleavage sites are detected and have >28 amino acids. The arrows indicate the cleavage sites that were exclusively detected in the rAra h 2 protein. The epitopes and cleavage sites are shown as described in the legend of Fig. 1.

Next, the peptides that were generated from the Ara h 2 protein following trypsin digestion were determined. A fragment with a molecular mass of 13.5 kDa was predominantly generated from nAra h 2 and rAra h 2 following 60-min trypsin digestion, and no further degradation of this fragment was observed. Moreover, this fragment had the same molecular weight as peptide A (**Fig. 5A**). Next, cleavage site mapping indicated that after trypsin digestion, the residual fragment was generated from the cleavage sites (R91) adjacent to the N-terminal amino acid residue (G93) of peptide A (**Fig. 5B, C**). Therefore, a fragment similar to peptide A was generated from nAra h 2 and rAra h 2 following trypsin digestion. A fragment identical to peptide A has been previously reported to be generated by trypsin digestion^20^^)^.

## 4. Discussion

**Fig. 6.**
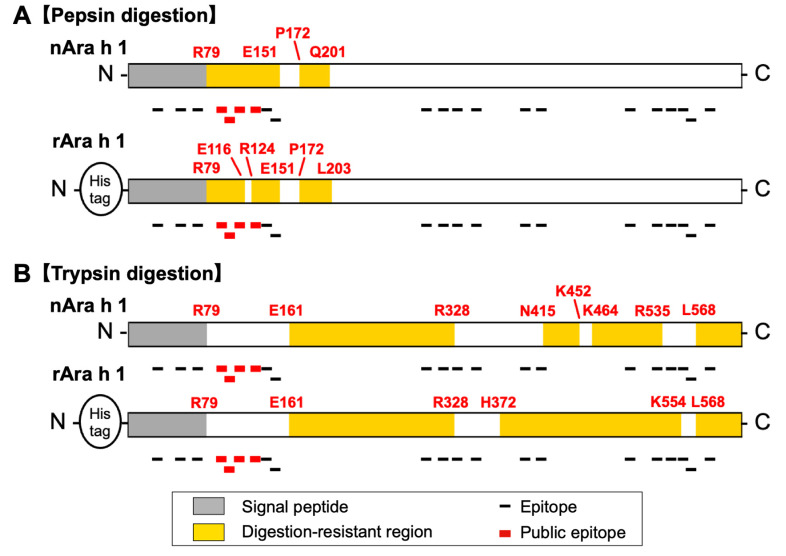
Diagrams of the digestion profiles of the Ara h 1 proteins generated from pepsin and trypsin digestion. (A) Diagram of the pepsin-digested Ara h 1 proteins. This figure is provided on the basis of the digestibility profile shown in Fig. 1B and C. (B) Diagram of the trypsin-digested Ara h 1 proteins. This figure is provided on the basis of the digestibility profile shown in Fig. 3B and C.

The deduced digestion-resistant and digestion-susceptible regions of Ara h 1 were provided, along with the location of its linear epitopes (**Fig. 6**). Overall, the pepsin digestion pattern of rAra h 1 closely resembled that of nAra h 1 (**Fig. 6A**). Upon closer examination, a region from R79 to E151 was found to be resistant to pepsin digestion in nAra h 1, whereas the corresponding region of rAra h 1 was fragmented at the cleavage sites E116 and R124. The quaternary structure of the Ara h 1 trimmer complex (PDB accession: 3SMH) is available. This ribbon model shows that an N-terminal region from M1 to T163 is excluded; therefore, the structure of the pepsin-digestion-resistant region from R79 to E151 cannot be determined. Another pepsin digestion-resistant region from P172 to Q201/L203 and a pepsin digestion-susceptible region from N441 to Y495/A497 are situated on the outer surface of the trimer complex (**Fig. S8**). Given the low pH of SGF, which can denature most proteins, providing accurate estimates of the relationship between pepsin digestibility and the structural characteristics of target proteins is difficult.

Moreover, the trypsin digestion patterns of nAra h 1 and rAra h 1 exhibited similar patterns (Fig. 6B). In particular, the region from N415 to R535 of rAra h 1 was resistant to trypsin digestion; this region was found to be fragmented at the K452 and K464 cleavage sites of the nAra h 1 protein. Therefore, the structural characteristics of the region adjacent to the region from K452 to K464 are different in rAra h 1 and nAra h1 (Figs. 6B and **S8**). Furthermore, the region from N415 to R535 of rAra h 1 exhibited resistance to trypsin digestion, but this region encompasses a sequence from F440 to Y495, which was susceptible to pepsin digestion. In general, different susceptibilities to pepsin and trypsin digestion may indicate that the acidic conditions related to SGF severely affect the tertiary structure of the target proteins.

**Fig. 7.**
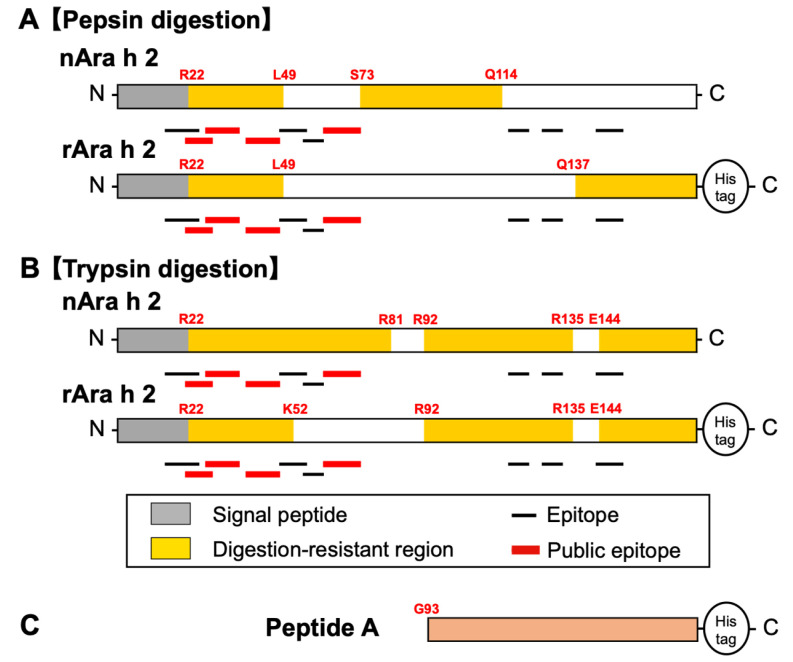
Diagrams of the digestion profiles of the Ara h 2 proteins generated from pepsin and trypsin digestion. (A) Diagram of the pepsin-digested Ara h 2 proteins. This figure is provided on the basis of the digestibility profile shown in Fig. 4E and F. (B) Diagram of trypsin-digested Ara h 2 proteins. This figure is provided on the basis of the digestibility profile shown in Fig. 5B and C.

The N-terminal region of Ara h 2 was resistant to pepsin digestion in nAra h 2 and rAra h 2. However, the C-terminal region exhibited distinct digestion patterns in these two proteins (Fig. 7A). This difference was attributed to nAra h 2-specific pepsin cleavage sites located at the C-terminal region (Fig. 4E). These cleavage sites were not detected in rAra h 2, thereby indicating that the His-tag extension at the C-terminus of rAra h 2 may affect pepsin cleavage in the C-terminal region. Nevertheless, other pepsin cleavage sites were commonly observed in Ara h 2 proteins (Fig. 4E, F). Compared with pepsin digestion, the patterns following trypsin digestion of rAra h 2 were broadly similar to those of nAra h 2 (Fig. 7B).

Peptide A, which appears to be unintentionally produced (Fig. 7C), exhibited exceptional resistance to pepsin digestion. Moreover, peptide A is identical to a peptide generated by trypsin digestion (Fig. 5A)^20^^)^. rAra h 2 was prepared from recombinant yeast, which indicates that peptide A is posttranslationally produced by proteolysis within yeast or may be produced from yeast cells during protein purification. Proteolytic degradation has been a challenge to the heterologous production of recombinant proteins because *P. pastoris* produces cytosolic proteases, vacuolar proteases, and extracellular proteases^31^^)^. Interestingly, a full-length rAra h 2 protein showed higher sensitivity to pepsin digestion than peptide A (Fig. 4A, B), indicating that the access of pepsin to peptide A was restricted (Fig. 7C). Notably, the reduction of the disulfide bonds present in peptide A resulted in the complete loss of pepsin insensitivity (Fig. 4D). Based on a previous study, Ara h 2 contained eight cysteine residues, resulting in four disulfide bonds, namely, C33-C116, C45-C103 (or C104), C104 (or C103)-C152, and C118-C160^29^^)^. These amino acid positions of the cysteine residues were altered from the reference^29^^)^ because the amino acid sequence shown in Fig. 4 includes the signal sequence. When the N-terminal region until R92 was removed and peptide A was generated, C116 and C103 (or C104), which previously formed a disulfide bond with C33 and C45, respectively, became a free cysteine residue. Then, a new disulfide bond between C103 (or 104) and C116 may form in peptide A. Consequently, the tertiary structure of peptide A may differ from that of the full-length Ara h 2 protein (**Fig. S9**).

The linear epitopes designated in the Allergen Database for Food Safety are indicated in the boxes and lines above the amino acid sequence (Figs. 1–5). Of these epitopes, peptides recognized by >30% of patients allergic to peanuts were referred to as public epitopes^32^^)^, which are indicated in red boxes and red lines (Figs. 1–5). The locations of these epitopes are also illustrated, with the epitopes and public epitopes shown in black and red bold lines, respectively (Figs. 6 and 7). The public epitopes were mapped to the N-terminal regions of Ara h 1 and Ara h 2, both of which are resistant to pepsin digestion. This observation indicates that indigestion of Ara h 1 and Ara h 2 in the gastric fluid may contribute to the development of peanut allergy.

In the digestion assay conducted for allergenicity assessment of NEPs, the impact of His-tags on the digestion resistance of the epitope-rich N-terminal region of Ara h 1 and Ara h 2 was found to be minimal. Therefore, the use of tagged recombinant proteins is considered to provide valuable information for safety evaluation. However, recombinant proteins may contain artifacts such as peptide A not found in their natural counterparts. Considering that these artifact proteins could affect digestibility outcomes, safety assessment against such artifacts should not be incorporated into the risk assessment of full-length recombinant proteins. It should be noted that other epitopes may potentially be identified in future studies, and there are inherent limitations in extrapolating these findings to distinct allergens from peanut.

## Supplementary materials
