# Supplementary material for: Digestibility of Natural and Recombinant Allergenic Peanut Proteins in Artificial Gastrointestinal Fluids
Source: Food Saf (Tokyo). 2025 Dec 19;13(4):78–90. doi: 10.14252/foodsafetyfscj.D-25-00016 (PMC12718106; doi:10.14252/foodsafetyfscj.D-25-00016)
Supplement: Supplementary file 1 [file foodsafetyfscj-13-4-78-s001.pdf]

Supplementary File

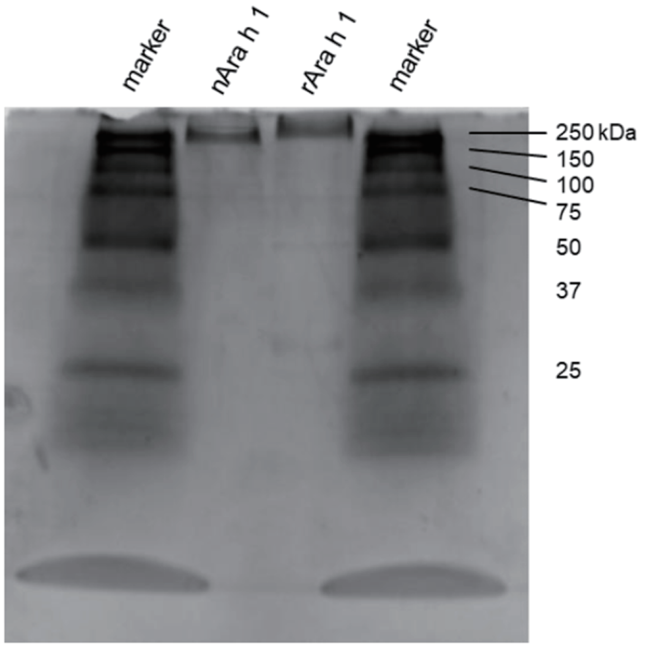

Fig. S1. Native-PAGE profile of the nAra h 1 and rAra h 1 proteins. Figure depicts a native-PAGE of the nAra h 1 and rAra h1 proteins. The separation was conducted using a 4% stacking and 15% resolving gel, with all procedures following a previously established protocol (see: http://www.assay-protocol.com/molecular-biology/electrophoresis/native-page.html).

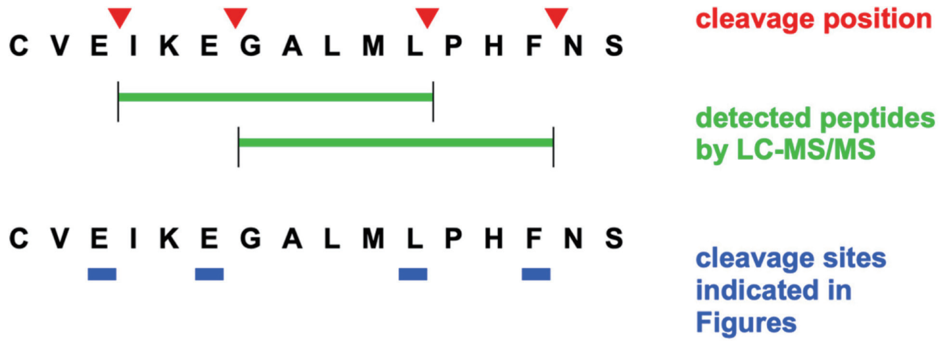

Fig. S2. Cleavage positions showing cleavage sites.
Shown are cleavage positions reported in Figures (see main text). Briefly, peptides detected by LC–MS/MS were mapped on the amino acid sequences of Ara h 1 and Ara h 2. The cleavage sites are indicated by short bars under the C-terminus amino acid sequences of the peptides generated by proteolysis.

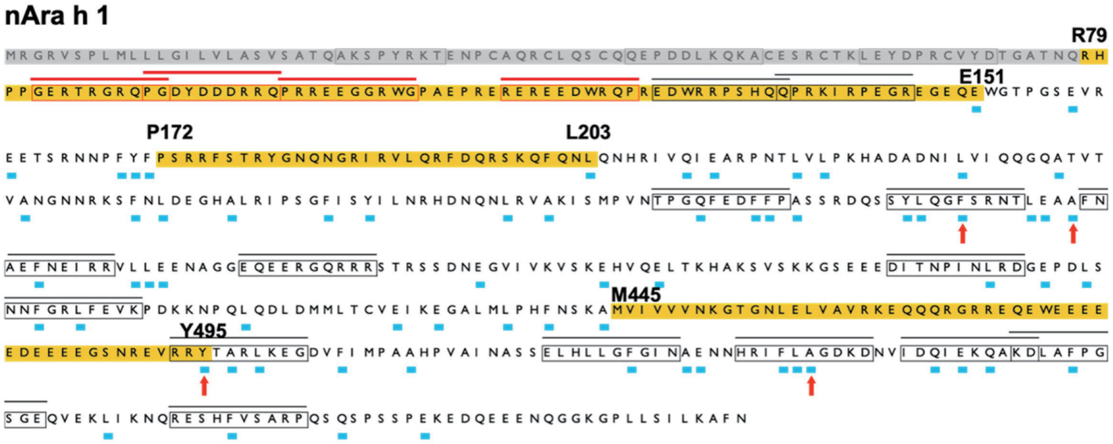

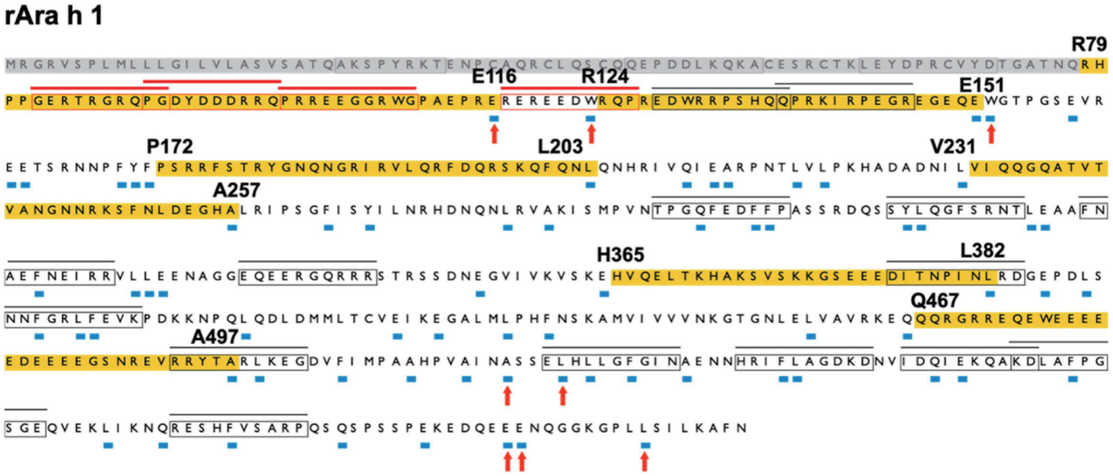

Fig. S3. Peptides generated by 5-min digestion of the Ara h 1 with S-pepsin.
Deduced cleavage sites were mapped onto the amino acid sequence of the Ara h 1 protein. The ratios of Ara h 1 to S-pepsin was 0.52:1. Amino acid sequences highlighted in yellow indicate the regions in which no cleavage sites were detected and have >28 amino acids. The amino acid sequences in red and black boxes indicate epitopes. To facilitate the understanding the epitope region, epitopes are also shown by red and black lines on the corresponding epitopes. Peptides shown in red boxes and red lines represent public epitopes. The arrows indicate cleavage sites that were exclusively detected in nAra h 1 and rAra h 1 proteins. These particular cleavage sites were inferred through comparative analysis of 5-min and 60-min digestion data.

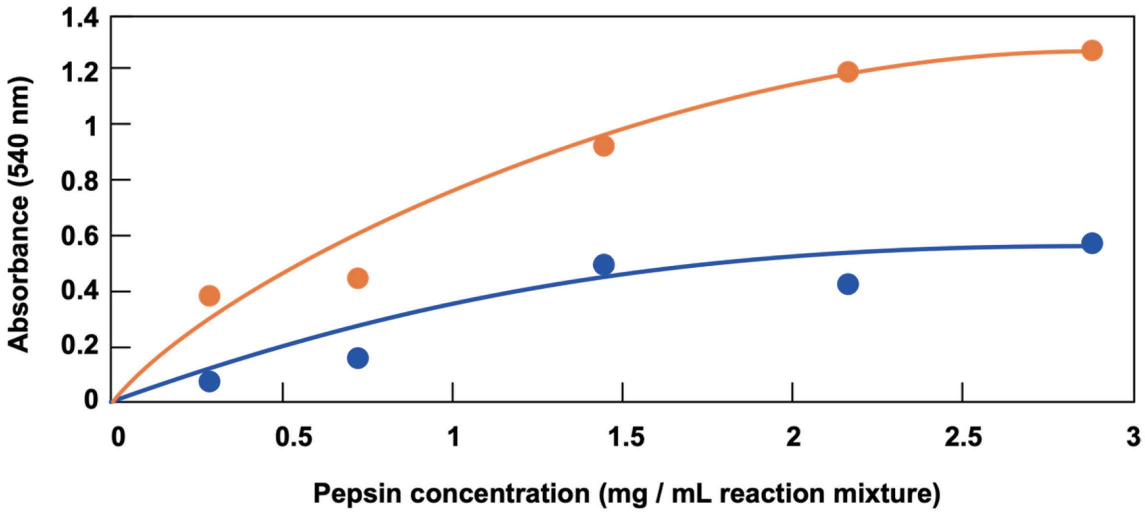

Fig. S4. Pepsin activity.
The activity of P-pepsin and S-pepsin is represented by orange and blue circles, respectively. The release of red dye from Azocoll was quantified as the protease activity.
Pepsin activity was quantified using Azocoll (Code No., 194932, Sigma-Aldrich) as its substrate (Ragster and Chrispeels, 1979). The reaction mixture consisted of 5-mg Azocoll, 900 µL SGF buffer, and 100 µL pepsin solution. The final concentrations of pepsin in the reaction mixture were adjusted to 0.3, 0.7, 1.4, 2.2, and 2.9 mg/mL, respectively. The mixture was incubated at 37°C with a 3-min vortexing interval for 20 min, after which the reaction was stopped by cooling on ice. Release of red dye from Azocoll was measured by measuring absorbance at 520 nm.

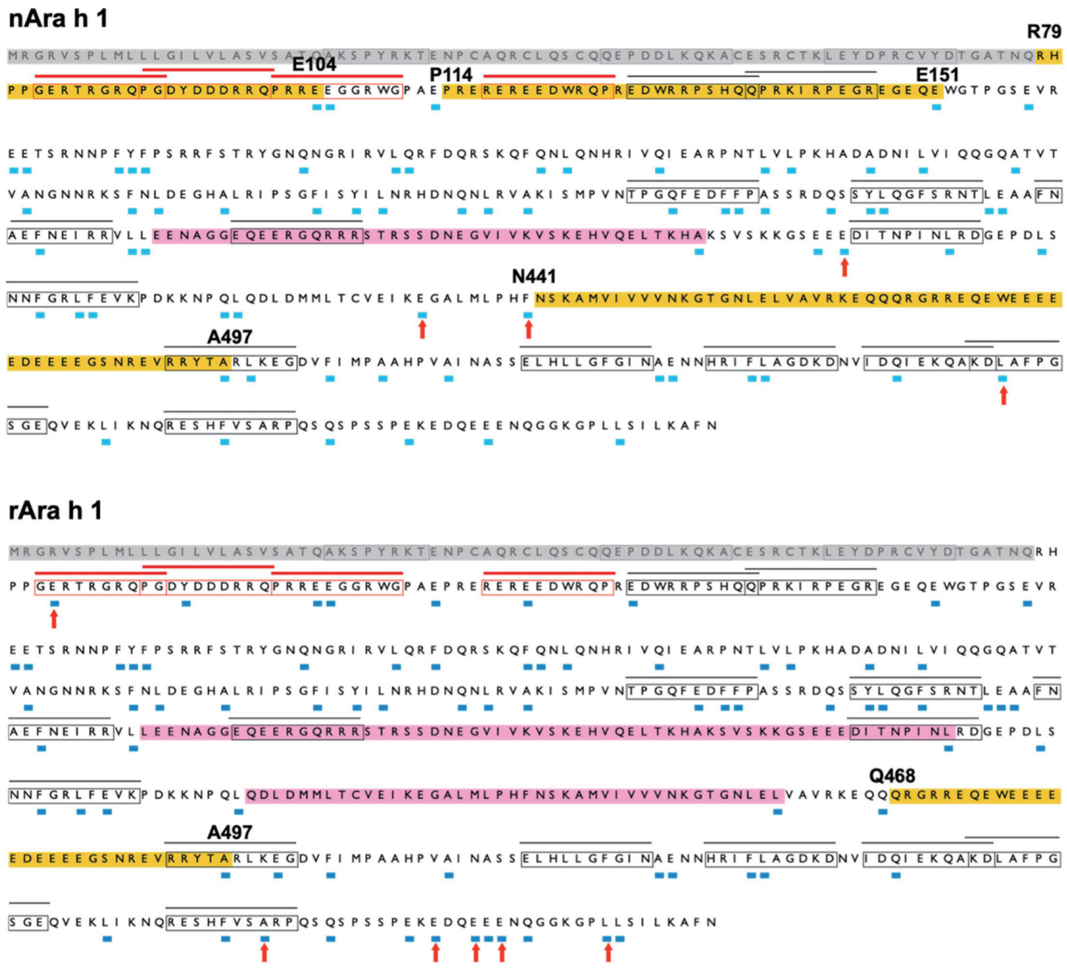

Fig. S5. Peptides generated by 60-min digestion of the Ara h 1 with P-pepsin.
Deduced cleavage sites were mapped onto the amino acid sequence of the Ara h 1 protein. The ratios of Ara h 1 to P-pepsin was 0.52:1. Amino acid sequences highlighted in yellow indicate regions in which no cleavage sites were detected and have >28 amino acids. Amino acid sequences highlighted in pink indicate regions in which no cleavage sites were present, as is the case with the yellow lines. However, multiple cleavage sites were detected in pink regions following analysis of peptides generated by 5-min digestion with P-pepsin. Therefore, the pink region indicates polypeptides that were completely degraded (i.e., to peptides smaller than 4 amino acids in length) following 60-min digestion. The arrows indicate cleavage sites that were exclusively detected in the nAra h 1 and rAra h 1 proteins. These cleavage sites were inferred via comparative analysis of 5-min and 60-min digestion data. Epitopes and cleavage sites are shown as described in the legend of **Figs. 1** and **S3**.

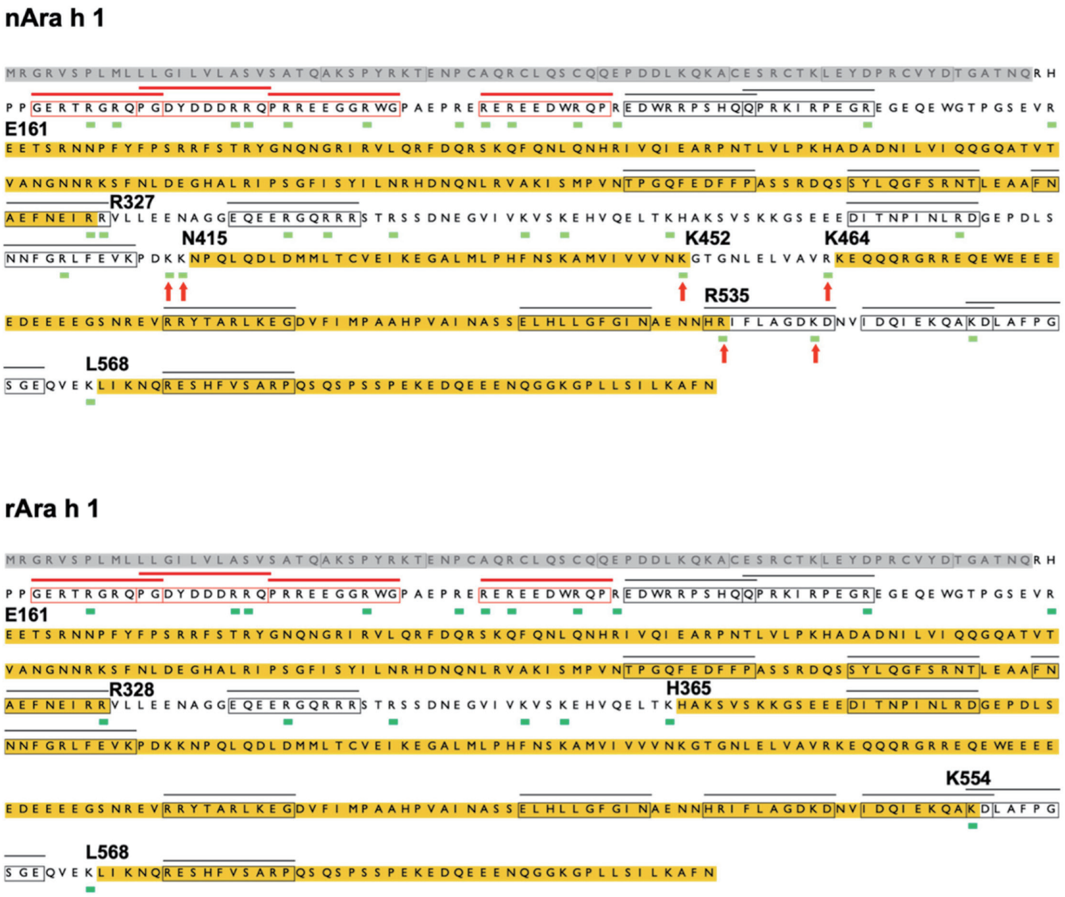

Fig. S6. Peptides generated by 120-min digestion of the Ara h 1 with trypsin.
Trypsin digestion of Ara h 1 protein was conducted in two independent replicates. The ratios of Ara h 1 to trypsin was 50:1. **Fig. 3** of the main text presents the results of one experiment. The results of the other digestion test is shown in this figure. Here, deduced cleavage sites were mapped onto the amino acid sequence of the Ara h 1 protein. Amino acid sequences highlighted in yellow indicate regions in which no cleavage sites were detected and have >28 amino acids. The arrows indicate cleavage sites that were exclusively detected in the nAra h 1 protein. These cleavage sites were inferred via comparative analysis of two duplicated datasets. Epitopes and cleavage sites are shown as described in the legend of **Figs. 1** and **S3**.

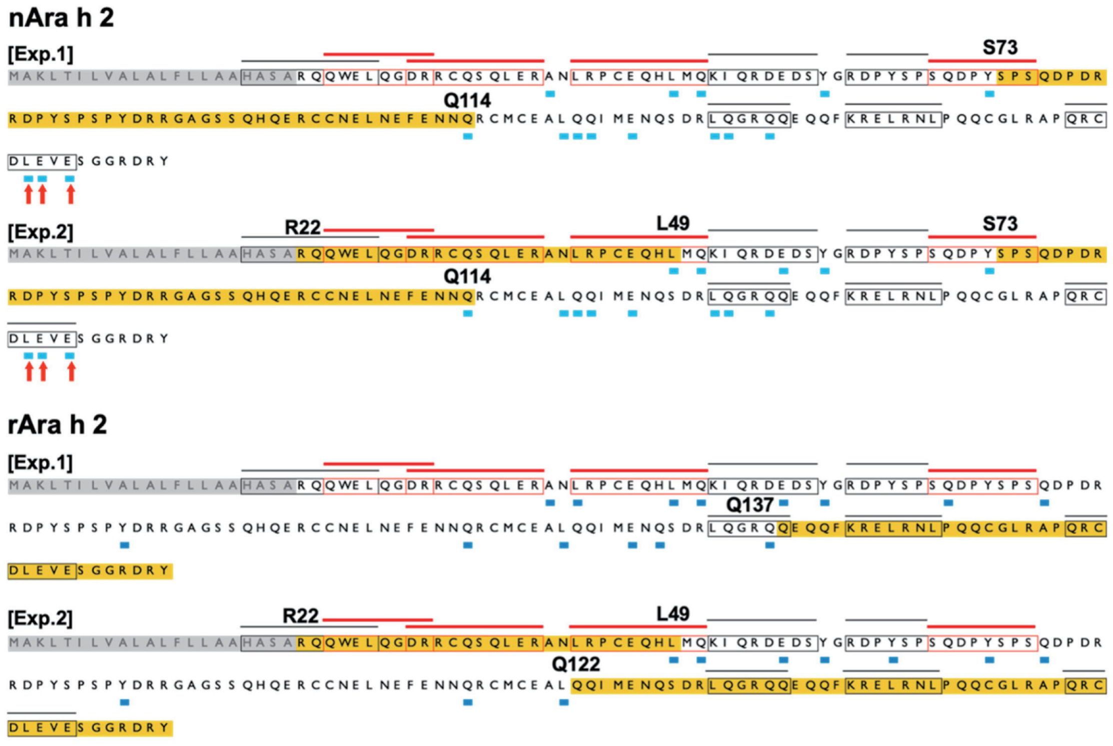

Fig. S7. Peptides generated by 60-min digestion of the Ara h 2 with P-pepsin.
Pepsin digestion of the Ara h 2 protein was conducted in two independent replicates. Here, deduced cleavage sites were mapped onto the amino acid sequence of the Ara h 2 protein. The ratio of Ara h 2 to P-pepsin was 0.052:1. Amino acid sequences highlighted in yellow indicate regions in which no cleavage sites were detected and have >28 amino acids. The arrows indicate cleavage sites that were exclusively detected in nAra h 2 proteins. These cleavage sites were inferred via comparative analysis of 5-min digestion and 60-min digestion datasets. Epitopes and cleavage sites are shown as described in the legend of **Figs. 1** and **S3**.

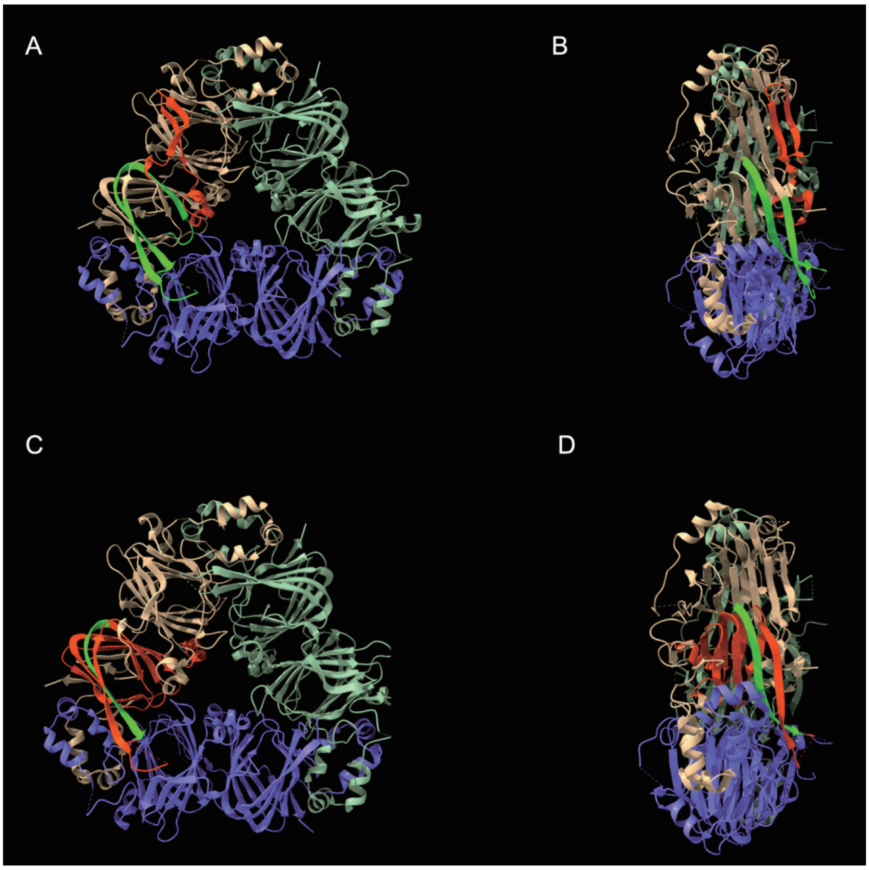

Fig. S8. Ribbon modeling of the trimer structure of Ara h 1.
The Ara h 1 crystal structure data (PDB accession: 3SMH) was edited using ChimeraX-1.6.1 software. This model encompasses S164 to P582 of the Ara h 1 protein (Cabanos et al., 2011).
(**A**), (**B**). The pepsin-digestion-resistant region from P172 to L203 and the pepsin-digestion-susceptible region from N441 to Y495 are shown in orange and light green ribbons, respectively.
(**C**), (**D**). The trypsin-digestion-resistant region from N415 to R535 is shown as an orange ribbon. The fragmented region from K452 to K464, which is specifically degraded in nAra h 1, is shown in light green.
Fig. B and **D** are rotated 90 degrees around the vertical axis of **Fig. A** and **C**, respectively.

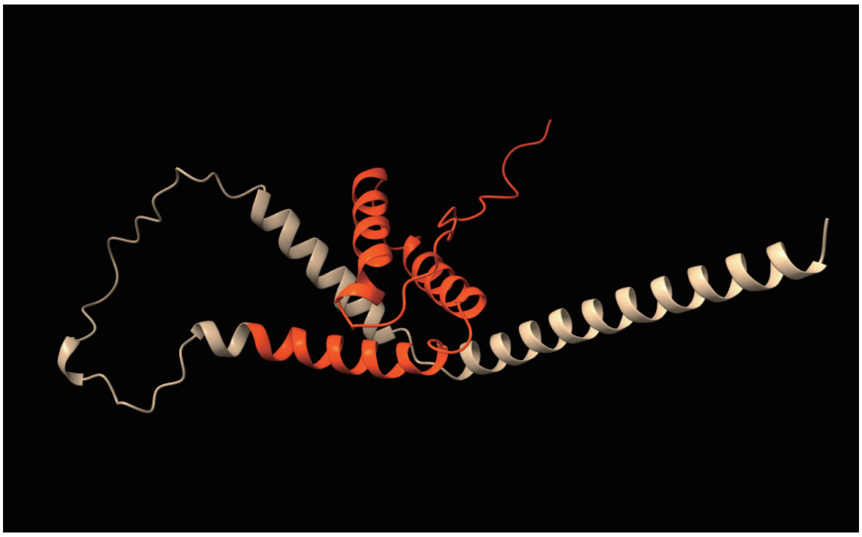

Fig. S9. Ribbon modeling of the Ara h 2 protein.
Ara h 2.01 predicted protein structure data (AlphaFold accession: AF-Q6PSU-F1-v4) edited using ChimeraX-1.6.1 software. The orange ribbon indicates peptide A (i.e., the region from G93 to Y172).

Table S1. The total number of distinct Ara h 1 peptides generated by proteolysis and the total number of cleavage sites observed.

Digestion conditions of Ara h 1	Number of peptides		Number of cleavage sites	
	nAra h 1	rAra h 1	nAra h 1	rAra h 1
S-pepsin 5 min	56	58	68	71
S-pepsin 60 min	71	67	75	80
S-pepsin 5min-Pancreatin 60 min	23	–	35	–
S-pepsin 5min-Pancreatin 120 min	32	–	50	–
P-pepsin 5 min	70	68	81	86
P-pepsin 60 min	58	62	72	76
Trypsin 120 min (Exp. 1)	18	17	30	23
Trypsin 120 min (Exp. 2)	24	12	31	18

Table S2. The total number of distinct Ara h 2 peptides generated by proteolysis and the total number of cleavage sites observed.

Digestion conditions of Ara h 2*	Number of peptides		Number of cleavage sites	
	nAra h 2	rAra h 2	nAra h 2	rAra h 2
P-pepsin 5 min (Exp. 1)	11	8	16	13
P-pepsin 5 min (Exp. 2)	10	6	15	10
P-pepsin 60 min (Exp. 1)	12	10	17	14
P-pepsin 60 min (Exp. 2)	12	7	17	10
Trypsin 120 min	3	4	5	7

* P-pepsin digestion was conducted at a Ara h 2:P-pepsin ratio of 0.052:1. See **Fig.4** of the main text.
